# Healthcare-associated infection prevention and control practices in Israel: results of a national survey

**DOI:** 10.1186/s12879-022-07721-8

**Published:** 2022-09-16

**Authors:** Ronza Najjar-Debbiny, Bibiana Chazan, Rona Lobl, M. Todd Greene, David Ratz, Sanjay Saint, Yehuda Carmeli, Mitchell J. Schwaber, the Israel IPC Working Group, Debby Ben-David, Pnina Shitrit, Alona Paz, Tal Brosh-Nissimov, Meirav Mor, Gili Regev-Yochay, Pnina Ciobotaro, Amos M. Yinnon, Dror Mar-Chaim, Bina Rubinovitch, Khetam Hussein, Shmuel Benenson

**Affiliations:** 1 Infection Prevention and Control Unit, Lady Davis Carmel Medical Center, Haifa, Israel; 2 Ruth and Bruce Rappaport Faculty of Medicine, Technion - Israel Institute of Technology, Haifa, Israel; 3 Infectious Disease Unit, Emek Medical Center, Afula, Israel; 4 National Center for Infection Control, Israel Ministry of Health, 6 Weizmann St., 6423906 Tel Aviv, Israel; 5 VA Ann Arbor Center for Clinical Management Research, VA Ann Arbor Healthcare System, Ann Arbor, MI USA; 6 Michigan Medicine Division of Hospital Medicine, University of Michigan, Ann Arbor, MI USA; 7 Tel Aviv University Sackler Faculty of Medicine, Tel Aviv, Israel

**Keywords:** Catheter-associated urinary tract infection, Central line-associated bloodstream infection, Ventilator-associated pneumonia, Hospital-acquired infection, Prevalence survey, COVID-19

## Abstract

**Background:**

Healthcare-associated infection (HAI) is a common and largely preventable cause of morbidity and mortality. The COVID-19 pandemic has presented unprecedented challenges to health systems. We conducted a national survey to ascertain hospital characteristics and the use of HAI prevention measures in Israel.

**Methods:**

We e-mailed surveys to infection prevention and control (IPC) leads of acute care hospitals in Israel. The survey included questions about the use of practices to prevent catheter-associated urinary tract infection (CAUTI), central line-associated bloodstream infection (CLABSI), ventilator-associated pneumonia (VAP), and *Clostridioides difficile* infection (CDI). The survey also assessed COVID-19 impact and healthcare worker well-being.

**Results:**

IPC leads from 15 of 24 invited hospitals (63%) completed the survey. Only one-third of respondents reported strong support for IPC from hospital leadership. Although several prevention practices were used by all hospitals (e.g., maximum sterile barrier precautions for CLABSI and real-time assessment of environmental cleaning for CDI), use of other practices was suboptimal—particularly for CAUTI and VAP. COVID-19 had a profound impact on Israeli hospitals, with all hospitals reporting opening of new units to care for COVID patients and most reporting moderate to extreme financial hardship. All hospitals reported highly successful plans to vaccinate all staff and felt confident that the vaccine is safe and effective.

**Conclusion:**

We provide a status report of the IPC characteristics and practices Israeli hospitals are currently using to prevent HAIs during the COVID-19 era. While many globally accepted IPC practices are widely implemented, opportunities to increase the use of certain IPC practices in Israeli hospitals exist.

**Supplementary Information:**

The online version contains supplementary material available at 10.1186/s12879-022-07721-8.

## Introduction

Healthcare-associated infection (HAI) is a major complication of medical therapy. Device-related infections such as catheter-associated urinary tract infection (CAUTI), central line-associated bloodstream infection (CLABSI), and ventilator-associated pneumonia (VAP) are among the most common healthcare-associated infections. Many of these HAIs are caused by multi-drug resistant organisms (MDRO), such as methicillin*-*resistant *Staphylococcus aureus* (MRSA), vancomycin-resistant enterococci (VRE), multidrug-resistant Gram-negative bacilli, and *Clostridioides difficile* infection (CDI) [1]. As HAIs are a leading cause of morbidity and mortality, further efforts are needed for their prevention and control [2].

Israel’s National Center for Infection Control, an arm of the Ministry of Health, spearheads infection prevention and control (IPC) efforts in healthcare institutions at the national level. By national mandate issued by the Ministry of Health, acute care hospitals must maintain IPC teams with designated staffing requirements. The IPC team is led by a physician specialist in infectious diseases and/or clinical microbiology (IPC lead), who devotes at least 50% of his or her time to IPC activities and reports directly to hospital administration. Additionally, the Ministry of Health annually offers performance-based monetary incentives to acute care hospitals to encourage compliance with high-priority IPC initiatives as designated by the National Center for Infection Control.

To our knowledge, prior studies have not extensively investigated the IPC structure and use of IPC practices in Israeli hospitals. Additionally, the COVID-19 pandemic has presented unprecedented new challenges to health systems and IPC efforts. We therefore conducted a nationwide cross-sectional survey to evaluate the use of currently recommended practices for preventing CAUTI, CLABSI, VAP, and CDI. We aimed as well to assess the impact that the ongoing COVID-19 pandemic has had on Israeli hospitals and healthcare worker well-being.

## Methods

### Study design and survey instrument

Data for this cross-sectional study were collected using a survey instrument based on the ‘Translating Healthcare-Associated Infection Prevention Research into Practice’ questionnaire developed by Krein, Saint, and colleagues [3–7] and previously distributed in the USA, Japan, Thailand, Switzerland, and the Netherlands [8–11]. The instrument contained questions about general hospital characteristics (e.g., number of beds), general infection prevention policies (e.g., presence of guidelines and surveillance systems), staffing and support of the infection control program, use of specific practices related to the prevention and monitoring of common healthcare-associated infections (CAUTI, CLABSI, VAP, and CDI), healthcare worker wellness, and the impact of the COVID-19 pandemic on hospitals and staff well-being.

In June 2021, IPC leads at all Israeli acute care hospitals with over 200 beds were invited by the National Center for Infection Control to participate. The questionnaire, with local adaptations made in consultation with the National Center for Infection Control, was distributed via e-mail to the IPC leads, to be completed using SurveyMonkey^®^ software (surveymonkey.com). The full survey is provided in Additional file 1.

### Statistical analysis

Descriptive statistics, n (%) for categorical and mean ± standard deviation (SD) for continuous variables were examined for all hospital characteristics as well as specific infection prevention practices. Responses about practice use were further categorized, with responses of 4 or 5 (i.e., ‘almost always use’ or ‘always use’) defined as regular use and coded as 1, and 0 otherwise. All statistical analyses were conducted in Stata (StataCorp. College Station, TX).

## Results

The IPC leads at 24 hospitals were invited to participate; 15 (63%) responded. A summary of individual responses is provided in Additional file 2. Fourteen participating facilities were general hospitals (6 tertiary care), and one was a pediatric tertiary care hospital. Select characteristics of participating hospitals are presented in Table 1. The mean acute care bed size was 615 beds, and all hospitals were affiliated with a medical school. Only one-third of respondents reported very good to excellent support for IPC teams from hospital leadership. All hospitals conducted hand hygiene surveillance via direct observation by a validated observer. The average overall reported compliance with hand hygiene directives was 83.1%. All hospitals reported having antimicrobial stewardship programs. Daily bathing with chlorhexidine gluconate was implemented in the ICU in most hospitals (92.9%), while this practice was implemented in only 21.4% of hospitals for non-ICU patients. Annual influenza vaccination for healthcare workers is mandatory in 1 of the 15 hospitals and encouraged in the other 14. Established surveillance was highest for CDI (hospital-wide in all 15 participating hospitals) and lowest for VAP (hospital-wide in only 1 of 14 responding hospitals—7%).

**Table 1:** Select hospital characteristics

	**% **or mean ± sd
Mean number of acute care hospital beds (including ICU beds)	614.5 ± 368.4
Mean reported hand hygiene compliance rate	83.1 ± 6.9%
Affiliated with a medical school	100%
Hospital epidemiologist on staff	73.3%
Good/excellent support from leadership for infection prevention	33.3%
Antimicrobial stewardship program	100%
Hand hygiene is very/extremely important priority	100%
Established surveillance system for monitoring CAUTI	86.7%
Established surveillance system for monitoring CLABSI	100%
Established surveillance system for monitoring VAP	42.9%
Established surveillance system for monitoring CDI	100%

*ICU* intensive care unit, *CAUTI* catheter-associated urinary tract infection, *CLABSI* central line-associated bloodstream infection, *VAP* ventilator-associated pneumonia, *CDI* *Clostridoides difficile* infection

The impact of the COVID-19 pandemic on hospitals is highlighted in Tables 2, 3. All hospitals reported opening new units and designated areas separated from other patients to care for COVID-19 patients. Most hospitals (73.3%) reported staffing shortages due to COVID-19 quarantine and isolation and 80% (based on 10 responses, as data missing for 5 of 15 responding hospitals) reported moderate to extreme financial hardship. All IPC leads believed that their hospital’s COVID-19 vaccination plan for the staff was effective, felt confident that the vaccine was safe and effective, and would receive the vaccine even if not required by their employer.

**Table 2:** COVID-19 response and challenges experienced in Israeli Hospitals

	**%**
Hospital has designated areas to care for COVID-19 patients that are separated from non-COVID patients	100%
Hospital has opened new units to care for COVID-19 patients	100%
Hospital has experienced staff shortages due to absences and/or illness during the COVID-19 pandemic	73.3%
Hospital pandemic response plan in addressing COVID-19 has been very/extremely effective	80.0%
Hospital has experienced moderate/extreme financial hardship resulting from the COVID-19 pandemic*	80.0%
Hospital COVID-19 vaccination plan has been very/extremely successful in vaccinating staff	100%
Hospital has experienced an increase in loss of staff (e.g., resignations) in the midst of COVID-19	40.0%

*No answer for 5 of the 15 responding hospitals. 80% based on the 10 responses

**Table 3:** COVID-19 response and challenges experienced by israeli infection preventionists

	**%**
Would (or already have) voluntarily receive COVID-19 vaccine, even if not required by employer	100%
Moderately/very confident that a COVID-19 vaccine is safe and effective	100%
Agree/strongly agree with the statement: “I feel safe carrying out my work role during the COVID-19 pandemic.”	93.3%

Factors describing IPC lead well-being and methods of promoting well-being are presented in Table 4. Burnout and feelings of becoming more uncaring were present but rare, with 2 (13%) IPC leads reporting feeling burned out from their work and 1 (7%) reporting feeling more uncaring towards people since taking their current job. Most IPC leads (80%) reported that spiritual well-being and individual self-care practices (such as yoga, meditation, and exercise) are important.

**Table 4:** Infection preventionist well-being

	**%**
I feel burned out from my work	13.3%
I have become more uncaring towards people since I took this job	6.7%
If given the opportunity to revisit my career choice, I would choose to become an infection preventionist again	73.3%
Spiritual well-being is important for one’s emotional well-being	80.0%
Religious or spiritual beliefs act as a source of comfort and strength during life’s ups and downs	60.0%
An organized religious or spiritual community is important to me	40.0%
Individual self-care practices (e.g., meditation, yoga, music, exercising, communing with nature) is important to me	80.0%

### Prevention and surveillance of device-related infections and CDI

IPC leads reported on hospital managements’ views regarding the importance of preventing HAI according to international standards. IPC leads perceived CLABSI prevention as important to management in 80% of participating hospitals, CDI prevention in 67%, CAUTI prevention in 33%, and VAP prevention in 20%.

**Fig. 1:**
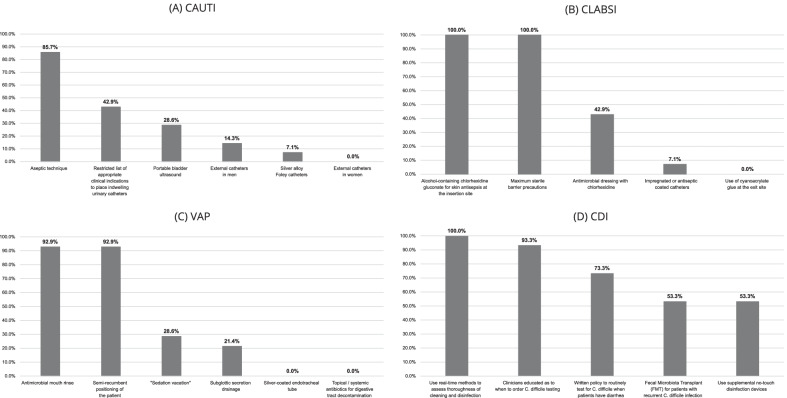
Infection Control Practices

CAUTI, CLABSI, VAP and CDI prevention practices in participating hospitals are summarized in Fig. 1. Some practices were universal, such as the use of sterile barriers for central line insertion and real-time assessment of environmental cleaning to prevent CDI. Other practices had low or no uptake, such as replacing indwelling catheters with intermittent catheterization for CAUTI prevention, and selective digestive tract decontamination for VAP prevention.

## Discussion

HAI is among the most serious adverse events that impact hospitalized patients. According to the World Health Organization, HAI affects an estimated 7.6% of patients in high-income countries [12]. Studies based on nation-wide surveys such as ours have been conducted in several countries, including the United States, Japan, Thailand, Switzerland, and the Netherlands, to quantify the use of accepted practices to prevent HAI [8, 11, 13–15]. To our knowledge, this is the first nation-wide study conducted in Israeli hospitals to describe the adherence to recommended practices used to prevent HAIs. Additionally, our survey was distributed during ongoing surges of COVID-19, permitting an unprecedented view of some of the impact that the pandemic has had on Israeli hospitals and IPC practices.

Several important findings on the use of specific recommended practices to prevent common HAIs emerged from our study. CAUTI prevention practices in Israel are sub-optimal. Aseptic technique during indwelling urethral catheter insertion and maintenance was the only CAUTI prevention practice regularly used by most Israeli hospitals. Only 13% of hospitals reported conducting facility-wide surveillance to monitor urinary tract infection, a proportion close to that reported in Swiss and Dutch hospitals [14], but far lower than the proportion previously reported in US (93.2%) [8] and Japanese (~ 50%) hospitals [13]. This low facility-wide reporting rate may be a function of a shortage of IPC staff, and absence of a national mandate to conduct CAUTI surveillance facility wide.

CLABSI prevention practices are in moderate to high use in Israeli hospitals. All responding hospitals reported full adherence to maximum sterile barrier precautions and use of solutions of alcohol and chlorhexidine gluconate for skin anti-sepsis at the insertion site. The adherence rate for these two recommended CLABSI prevention practices [16–18] was also universal in US and Dutch hospitals [11]. Although maximum sterile barrier precautions were also regularly used in Japan and Switzerland, the use of chlorhexidine gluconate for skin anti-sepsis at the insertion site was used in only approximately half of Japanese and Swiss hospitals. The moderate to high use of CLABSI prevention practices in Israel may be in part attributable to the inclusion of CLABSI prevention outcomes in the IPC incentive program conducted by the Ministry of Health, prompting hospital leadership to prioritize this in setting institutional IPC policy.

VAP prevention practices are generally not widespread in Israel, with the exception of semi-recumbent positioning and mouth rinse, each performed in over 90% of hospitals. The percentage of hospitals performing antimicrobial mouth rinse is similar to that reported in US hospitals and higher than reported in Swiss and Japanese hospitals [11].

Of note, at the time the survey was conducted antimicrobial mouth rinse was still an accepted element of VAP prevention bundles. Since then, updated Compendium VAP prevention guidelines have been published, which no longer recommend antimicrobial mouth rinse as a preventive measure [19]. We note also that VAP prevention and surveillance are not nationally mandated in Israel, but are rather prioritized and managed at the institutional level, at the discretion of hospital management.

CDI surveillance was conducted in all responding Israeli hospitals and use of all CDI prevention practices was moderate to high, consistent with reported use in US, Dutch, and Swiss hospitals [11]. Guidelines indicate that the combination of contact precautions (strongest level of evidence) and rigorous hand hygiene is most effective in CDI prevention [20]. All responding Israeli hospitals reported using real-time methods to assess thoroughness of cleaning and disinfection of environmental surfaces in patient rooms, and reported hand hygiene compliance was approximately 85%. Additionally, all Israeli hospitals reported having antimicrobial stewardship programs. Taken together, prioritization of CDI prevention practices in Israeli hospitals is strong. Improving diagnostic stewardship when testing for CDI is a primary approach for reducing CDI in acute care settings [21]. Although nearly all Israeli hospitals reported that clinicians were educated regarding when to order CDI testing, further promoting written policies for routine testing for CDI among patients with diarrhea in Israeli hospitals is encouraged.

The COVID-19 pandemic has placed substantial burdens on healthcare systems and hospitals worldwide. Hospitals in Israel were no exception, with all hospitals responding to our survey reporting space and logistical demands, staffing issues, and financial hardship. Still, Israeli IPC leads reported a relatively low percentage of burnout compared to percentages reported among healthcare workers in other countries, despite the ongoing surges of the COVID-19 pandemic [22, 23]. Although not specifically linked in the questionnaire, it is possible that the importance placed by the majority of respondents on spiritual well-being and individual self-care practices is related to the relatively low percentage of burnout that we found.

Faith in and support of COVID-19 vaccinations were very strong in Israeli hospitals, with all IPC leads reporting a willingness to receive the vaccination and confidence that the vaccine is both safe and effective. Furthermore, all but one of the responding IPC leads indicated feeling safe performing their work roles during the pandemic. These findings align with the fact that Israel has been at the forefront of promoting and globally advocating for COVID-19 vaccination since the early phases of the pandemic.

Our findings indicate the importance of national IPC mandates, supplemented in certain cases by inclusion in an incentive program to encourage compliance. Where there is a national mandate (e.g., hospital-wide surveillance of CDI; CLABSI surveillance in ICUs), uniform compliance was reported. In addition, CLABSI prevention in ICUs is incentivized, including monetarily, by Ministry of Health programming, perhaps contributing to high compliance with prevention measures in these units. By contrast, there is no national requirement to conduct VAP surveillance, and the requirement for CAUTI surveillance is limited to specific wards and specific times of the year. Neither CAUTI nor VAP outcomes are rewarded by inclusion in the IPC incentive program. These factors may explain in part the relatively low adherence to recognized preventive measures for these infections, in comparison to those observed in other countries and notwithstanding their inclusion in global IPC standards. Further exploration of the roles of both national mandates and monetary incentives to achieve compliance with accepted IPC practices is warranted.

While our study was nationwide and thus broadly encompassing, several limitations must be acknowledged. First, we relied entirely on self-reporting from the IPC lead at each hospital to determine the practices used to prevent HAIs, potentially leading to information bias. Second, the study was conducted in the midst of the COVID-19 pandemic, which may have influenced IPC attitudes, capabilities and practices. Third, surgical site infections and other infection prevention topics were not covered in this survey. Fourth, as this is the first nationwide study of its kind in Israel, national comparative data over time are lacking. Finally, our survey did not assess the incidence of the various HAIs at each hospital. Although we are therefore unable to link observed infection prevention process measures with HAI outcomes, future studies may explore these relationships.

## Conclusion

Our study is, to our knowledge, the first national assessment of HAI prevention practices in Israeli hospitals and provides a unique snapshot of IPC practices during the COVID-19 pandemic. Although Israeli acute care hospitals are using many recommended HAI prevention practices, there are opportunities to improve the adoption and regular use of certain practices—particularly for CAUTI and VAP prevention. To further improve the adoption of key infection prevention practices among Israeli hospitals, hospital-wide implementation strategies and infection prevention prioritization are needed. Expanding current IPC mandates and incentive structures through the Ministry of Health is a potential mechanism for improving and maintaining optimal infection prevention practices in Israeli hospitals.

## Supplementary Information

**Additional file 1.** Survey content as used in this study.

**Additional file 2. **Results for individual survey questions.

## Data Availability

The datasets used and analyzed in the context of this survey are available from the corresponding author upon reasonable request.

## Abbreviations

CAUTI: Catheter-associated urinary tract Infection
CDI: *Clostridioides difficile* Infection
CLABSI: Central line-associated bloodstream infection
HAI: Healthcare-associated infection
ICU: Intensive care unit
VAP: Ventilator-associated pneumonia
