# Supplementary material for: Healthcare-associated infection prevention and control practices in Israel: results of a national survey
Source: BMC Infect Dis. 2022 Sep 16;22:739. doi: 10.1186/s12879-022-07721-8 (PMC9482296; doi:10.1186/s12879-022-07721-8)
Supplement: Supplementary file 1 — Additional file 1. Survey content as used in this study. [file 12879_2022_7721_MOESM1_ESM.docx]

Supplementary Appendix 1: Survey

Healthcare-associated infection prevention and control practices in Israel: Results of a national survey

Questions about your Hospital:

1. Total number of adult acute care beds at your hospital? _________
2. Total number of adult Intensive Care Unit (ICU) beds at your hospital (please include all medical, surgical, neurosurgical, cardiothoracic, and cardiac)? _________
3. Approximately what percentage of your rooms are currently:
   1. Private (1 patient) _______ %
   2. Non-private (2 or more patients) _______ %
4. Is your hospital affiliated with a medical school?

_1_ No

_2_ Yes

_3_ Don’t know

1. At your hospital, does the highest-ranking physician (e.g., Chief Medical Officer, Chief of Staff) provide direct patient care?

_1_ No

_2_ Yes

_3_ Don’t know

1. At your hospital, does the highest-ranking nurse (e.g., Chief Nurse, Director of Patient Care Services) provide direct patient care?

_1_ No

_2_ Yes

_3_ Don’t know

Questions about your infection control program:

**Here are some definitions to assist you when answering the following questions:**

**Hospital Epidemiologist:** MD, PhD, or RN with expertise in hospital epidemiology and infection control.

**Infection Preventionist:** Individual, other than the Hospital Epidemiologist, responsible for the management and day-to-day activities of the Infection Control Program (with or without Certification in Infection Control).

1. How would you rank the overall support (e.g., staffing, financial, and political) your infection prevention and control program receives from the hospital administrative leadership?

_1_ Poor

_2_ Fair

_3_ Good

_4_ Very Good

_5_ Excellent

1. Does your hospital have a Hospital Epidemiologist?

_1_ No

_2_ Yes

_3_ Don’t know

#### Questions about the Infection Preventionists at your hospital:

1. How many Infection Preventionists does your Infection Control Program have?
   1. ___________ Full-time
   2. ___________ Part-time
2. The following pertain only to the person completing this survey. For each of the statements below please indicate the extent of your **agreement or disagreement** by checking the appropriate box.

|  | **Strongly disagree** | **Disagree** | **Neither disagree nor agree** | **Agree** | **Strongly agree** |
| --- | --- | --- | --- | --- | --- |
| A. I feel burned out from my work. | _1_ | _2_ | _3_ | _4_ | _5_ |
| B. I have become more uncaring towards people since I took this job. | _1_ | _2_ | _3_ | _4_ | _5_ |
| C. If given the opportunity to revisit my career choice, I would choose to become an infection preventionist again. | _1_ | _2_ | _3_ | _4_ | _5_ |
| D. Spiritual well-being is important for one’s emotional well-being. | _1_ | _2_ | _3_ | _4_ | _5_ |
| E. Religious or spiritual beliefs act as a source of comfort and strength during life’s ups and downs. | _1_ | _2_ | _3_ | _4_ | _5_ |
| F. An organized religious or spiritual community is important to me. | _1_ | _2_ | _3_ | _4_ | _5_ |
| G. Individual self-care practices (e.g., meditation, yoga, listening to music, exercising, communing with nature) is important to me. | _1_ | _2_ | _3_ | _4_ | _5_ |

1. The following questions ask about your views of the hospital where you work. For each of the statements below please indicate the extent of your **agreement or disagreement** by checking the appropriate box.

|  | **Strongly disagree** | **Disagree** | **Neither disagree nor agree** | **Agree** | **Strongly agree** |
| --- | --- | --- | --- | --- | --- |
| A. I assert my views on important issues, even though my supervisor may disagree. | _1_ | _2_ | _3_ | _4_ | _5_ |
| B. I personally feel comfortable speaking up when I see a physician not clean his or her hands. | _1_ | _2_ | _3_ | _4_ | _5_ |
| C. When a medical error occurs at this hospital, employees are encouraged to discuss mistakes in order to learn how to prevent similar future errors. | _1_ | _2_ | _3_ | _4_ | _5_ |
| D. Leadership is driving us to be a safety-centered institution. | _1_ | _2_ | _3_ | _4_ | _5_ |
| E. I would feel safe being treated here as a patient. | _1_ | _2_ | _3_ | _4_ | _5_ |
| F. If you make a mistake at this hospital, it is often held against you. | _1_ | _2_ | _3_ | _4_ | _5_ |
| G. Employees at this hospital are able to bring up problems and tough issues. | _1_ | _2_ | _3_ | _4_ | _5_ |
| H. It is safe to try something new at this hospital. | _1_ | _2_ | _3_ | _4_ | _5_ |
| I. At this hospital, people are too busy to invest time in improvement. | _1_ | _2_ | _3_ | _4_ | _5_ |
| J. In this hospital, employees are expected to question leadership. | _1_ | _2_ | _3_ | _4_ | _5_ |
| K. In this hospital, authority is concentrated at the top. | _1_ | _2_ | _3_ | _4_ | _5_ |

# *If you happen to have both acute and long-term care beds at your hospital please answer the remaining survey questions only for adult acute care:*

# *Catheter-Associated Urinary Tract Infection:*

1. The following pertain to adults in your acute care hospital who require **urinary collection and/or urinary output monitoring**.

| **Practices to prevent catheter-associated urinary tract infection** | Using a scale from 1 to 5  (1 being never and 5 being always), please indicate **how** **frequently the practice is used** in your hospital. |
| --- | --- |
| A. Portable bladder ultrasound scanner for determining post-void residual | 1 2 3 4 5  Never Always |
| B. Urinary catheter reminder or stop-order | 1 2 3 4 5  Never Always |
| C. Nurse-initiated urinary catheter discontinuation | 1 2 3 4 5  Never Always |
| D. Silver alloy Foley catheters | 1 2 3 4 5  Never Always |
| E. External catheters in men (e.g., condom catheters, glans-adherent devices) | 1 2 3 4 5  Never Always |
| F. External catheters in women (e.g., wicking devices such as PureWick^™^ and PrimaFit®) | 1 2 3 4 5  Never Always |
| G. Aseptic technique during indwelling urethral catheter insertion and maintenance | 1 2 3 4 5  Never Always |
| H. Intermittent catheterization | 1 2 3 4 5  Never Always |
| I. Restricted list of appropriate clinical indications to place indwelling urinary catheters | 1 2 3 4 5  Never Always |

1. Does your hospital have a system for monitoring which patients have urinary catheters placed?

_1_ No

_2_ Yes, but only in some units (e.g., ICU)

_3_ Yes, hospital wide

_4_ Don’t know

1. Where are the **majority** of indwelling urinary catheters placed at your hospital? (select only one)

_1_ Emergency Department

_2_ Operating Room

_3_ Floor (including telemetry/step down)

_4_ Intensive Care Unit

_5_ Other, please specify: ____________________________________

1. Does your hospital conduct daily rounds to assess the ongoing necessity of indwelling urinary catheters?

_1_ No

_2_ Yes, but only in some units (e.g., ICU)

_3_ Yes, hospital wide

_4_ Don’t know

1. Does your hospital routinely monitor duration and/or discontinuation of urinary catheters?

_1_ No

_2_ Yes, but only in some units (e.g., ICU)

_3_ Yes, hospital wide

_4_ Don’t know

1. Does your hospital have an established surveillance system for monitoring urinary tract infection rates?

_1_ No

_2_ Yes, but only in some units (e.g., ICU)

_3_ Yes, hospital wide

_4_ Don’t know

1. Does your hospital report urinary tract infection rates to direct care providers?

_1_ No

_2_ Yes, but only in some units (e.g., ICU)

_3_ Yes, hospital wide

_4_ Don’t know

1. Does your hospital report urinary catheter utilization ratios to direct care providers?

_1_ No

_2_ Yes, but only in some units (e.g., ICU)

_3_ Yes, hospital wide

_4_ Don’t know

1. What is your perception of how important it is to hospital leadership at your hospital to prevent urinary tract infections?

_1_ Minimally important

_2_ Moderately important

_3_ Very important

_4_ Extremely important

1. Does your hospital perform routine urine tests (for example, urinalysis and/or urine culture) to screen for urinary tract infection at the time of hospital admission?

_1_ No

_2_ Yes

_3_ Don’t know

1. Does your hospital use any of the following strategies to reduce inappropriate urine testing (i.e., diagnostic stewardship)? (select all that apply)

_1_ Require indication when placing order for urine test

_2_ Preserving urine cultures in emergency room for up to 48 hours and only processing if requested by emergency room physician after clinical assessment

_3_ Encourage appropriate urine testing using decision-support or electronic alerts (e.g., best practice alerts)

_4_ Rejecting contaminated urine specimens

_5_ Hiding entire urine culture results and releasing only at provider request

_6_ Selective suppression of antimicrobial susceptibility results

_7_ Inclusion of wording or phrasing in the urine culture results to remind clinicians not to treat asymptomatic bacteriuria

_8_ Reflex urine testing (i.e., only perform urine culture when urinalysis is positive)

_9_ Nurse-directed education/initiative to reduce urine testing

_10_ Remove/limit urine culture testing

# *Central Venous Catheter-Related Infection:*

1. The following pertain to adults hospitalized in your facility who require **short-term** **central venous catheters**.

| **Practices to prevent central venous catheter-related infection** | Using a scale from 1 to 5  (1 being never and 5 being always), please indicate **how** **frequently the practice is used** in your hospital. |
| --- | --- |
| A. Maximum sterile barrier precautions (full gown, sterile gloves, full body sterile drape) during central catheter insertion | 1 2 3 4 5  Never Always |
| B.  Alcohol-containing chlorhexidine gluconate for skin antisepsis at the insertion site | 1 2 3 4 5  Never Always |
| C.  Advanced securement device (e.g., Tegaderm™ IV Advanced, SecurAcath®) | 1 2 3 4 5  Never Always |
| D.  Impregnated or antiseptic coated catheters | 1 2 3 4 5  Never Always |
| E.  Use of cyanoacrylate glue at the exit site | 1 2 3 4 5  Never Always |
| F.  Antimicrobial dressing with chlorhexidine (Biopatch™) | 1 2 3 4 5  Never Always |
| G. Restricted list of appropriate clinical indications to place central venous catheters | 1 2 3 4 5  Never Always |

1. Does your hospital have an established surveillance system for monitoring central venous catheter-related infection rates?

_1_ No

_2_ Yes, but only in some units (e.g., ICU)

_3_ Yes, hospital wide

_4_ Don’t know

1. Does your hospital report central venous catheter-related infection rates to direct care providers?

_1_ No

_2_ Yes, but only in some units (e.g., ICU)

_3_ Yes, hospital wide

_4_ Don’t know

1. Who is responsible for inserting the **majority** of **non-peripherally inserted central venous catheters** at your hospital? (select only one)

_1_ Designated Vascular Access Nurse or Nursing Team

_2_ Interventional Radiologists

_3_ Hospitalists

_4_ Emergency Room Physicians

_5_ Intensive Care Unit Physicians

_6_ Respiratory Therapists

_7_ Other, please specify: ____________________________________

1. Does your hospital have a list of indications to determine the appropriateness of central venous catheters prior to placement?

_1_ No

_2_ Yes

_3_ Don’t know

1. Who is responsible for inserting the **majority** of **peripherally inserted central catheters (PICCs)** at your hospital?

_1_ Designated Vascular Access Nurse or Nursing Team

_2_ Interventional Radiologists

_3_ Hospitalists

_4_ Emergency Room Physicians

_5_ Intensive Care Unit Physicians

_6_ Respiratory Therapists

_7_ Other, please specify: ____________________________________

1. Does your hospital have a list of indications to determine the appropriateness of PICCs prior to placement?

_1_ No

_2_ Yes

_3_ Don’t know

1. Does your hospital use appropriateness guidelines (e.g., Michigan MAGIC) for PICC use?

_1_ No

_2_ Yes

_3_ Don’t know

1. Does your hospital conduct daily rounds to assess the ongoing necessity of PICCs?

_1_ No

_2_ Yes, but only in some units (e.g., ICU)

_3_ Yes, hospital wide

_4_ Don’t know

1. What is your perception of how important it is to hospital leadership at your hospital to prevent central venous catheter-related infections?

_1_ Minimally important

_2_ Moderately important

_3_ Very important

_4_ Extremely important

***Ventilator-Associated Events:***

1. The following questions pertain to adults in your acute care hospital who require **mechanical ventilation**.

| **Practices to prevent ventilator-associated events** | Using a scale from 1 to 5  (1 being never and 5 being always), please indicate **how** **frequently the practice is used** in your hospital. |
| --- | --- |
| A. Semi-recumbent positioning of the patient (head of bed elevated 30 degrees or more) | 1 2 3 4 5  Never Always |
| B. Antimicrobial mouth rinse (e.g., Peridex®) | 1 2 3 4 5  Never Always |
| C. Subglottic secretion drainage (via a special endotracheal tube) | 1 2 3 4 5  Never Always |
| D. Topical and/or systemic antibiotics for selective digestive tract decontamination | 1 2 3 4 5  Never Always |
| E. Silver-coated endotracheal tube | 1 2 3 4 5  Never Always |
| F. “Sedation vacation” (e.g., regular interruption of sedation) | 1 2 3 4 5  Never Always |

1. Does your hospital encourage early mobilization of ventilated patients as a strategy to prevent ventilator-associated events?

_1_ No

_2_ Yes, but only in some units (e.g., ICU)

_3_ Yes, hospital wide

_4_ Don’t know

1. Does your hospital have an established surveillance system for monitoring ventilator-associated event rates?

_1_ No

_2_ Yes, but only in some units (e.g., ICU)

_3_ Yes, hospital wide

_4_ Don’t know

1. Does your hospital report ventilator-associated event rates to direct care providers?

_1_ No

_2_ Yes, but only in some units (e.g., ICU)

_3_ Yes, hospital wide

_4_ Don’t know

1. What is your perception of how important it is to hospital leadership at your hospital to prevent ventilator-associated events?

_1_ Minimally important

_2_ Moderately important

_3_ Very important

_4_ Extremely important

***Clostridioides difficile Infection:***

1. Does your hospital use supplemental no-touch disinfection devices for rooms used to care for patients with *C. difficile* infection (e.g., ultraviolet germicidal irradiation, hydrogen peroxide vapor)?

_1_ No

_2_ Yes, but only in some units (e.g., ICU)

_3_ Yes, hospital wide

_4_ Don’t know

1. Does your hospital use real-time methods to assess thoroughness of cleaning and disinfection of environmental surfaces in patient rooms (e.g., fluorescent marker, adenosine triphosphate (ATP) testing)?

_1_ No

_2_ Yes, but only in some units (e.g., ICU)

_3_ Yes, hospital wide

_4_ Don’t know

1. Does your hospital have a written policy to routinely test for C. difficile when patients have diarrhea while on antibiotics or within several months of taking them?

_1_ No

_2_ Yes, but only in some units (e.g., ICU)

_3_ Yes, hospital wide

_4_ Don’t know

1. Does your hospital have an established surveillance system for monitoring *C. difficile* infection rates?

_1_ No

_2_ Yes, but only in some units (e.g., ICU)

_3_ Yes, hospital wide

_4_ Don’t know

1. Are clinicians at your hospital educated as to when to order *C. difficile* testing?

_1_ No

_2_ Yes

_3_ Don’t know

1. Which test does your laboratory primarily use to determine the presence of *C. difficile*? (select only one)

_1_ Antigen enzyme immunoassay

_2_ Toxin enzyme immunoassay

_3_ Polymerase chain reaction (PCR)

_4_ Culture/Cytotoxin assay

_5_ Some combination of the above

1. Does your hospital use any of the following strategies to reduce inappropriate testing for *C. difficile* (i.e., diagnostic stewardship)? (select all that apply)

_1_ Reject formed stool submitted for *C. difficile* testing

_2_ Reject testing for *C. difficile* on patients who have received laxatives within last 72 hours

_3_ Discourage testing for *C. difficile* on patients on laxatives using decision-support or electronic alerts (e.g., best practice alerts)

_4_ Use of cascade testing or hiding test results

_5_ Other, please specify: ______________________________________________________________

1. Does your hospital offer Fecal Microbiota Transplant (FMT) for patients with recurrent *C. difficile* infection?

_1_ No

_2_ Yes

_3_ Don’t know

1. Does your hospital report *C. difficile* infection rates to direct care providers?

_1_ No

_2_ Yes, but only in some units (e.g., ICU)

_3_ Yes, hospital wide

_4_ Don’t know

1. What is your perception of how important it is to hospital leadership at your hospital to prevent *C. difficile* infection?

_1_ Minimally important

_2_ Moderately important

_3_ Very important

_4_ Extremely important

# *General Infection Prevention Practices:*

1. Do you feel you are under any pressure to NOT report healthcare-associated infections at your hospital?

_1_ No

_2_ Yes

_3_ Don’t know

1. Among all patient safety issues, how important is hand hygiene at your hospital?

_1_ Minimally important

_2_ Moderately important

_3_ Very important

_4_ Extremely important

1. What was the last overall hand hygiene compliance rate reported in your hospital (between 0 and 100%)?

_______ %

1. Which surveillance method(s) does your hospital use for monitoring hand hygiene compliance (select all that apply)?

_1_ Direct observation method by validated observer

_2_ Calculation of hand hygiene product consumption

_3_ Electronic system

_4_ Self-report by staff

_5_ Other, please specify: ________________________________________________

1. Does your hospital have an antimicrobial stewardship program?

_1_ No (If no, skip to question 57)

_2_ Yes

_3_ Don’t know

1. If yes, please indicate who is on your antimicrobial stewardship team (select all that apply).

_1_ Infection Preventionist _5_ Pharmacist (**with** infectious diseases training)

_2_ Infectious Diseases Physician _6_ Pharmacist (**without** **or unknown** infectious diseases training)

_3_ Hospitalist _7_ Nurse

_4_ Other Physician _8_ Other, please specify: _______________________________

1. Please indicate below if your antimicrobial stewardship program measures aggregate antibiotic use (e.g., defined daily dose, days of therapy) for use in any of the following settings: (select all that apply)

_1_ Inpatient units (in hospital)

_2_ Outpatient/ambulatory clinics associated with your hospital

_3_ At the time of discharge from the hospital

_4_ Emergency room(s)

_5_ Urgent care facilities associated with your hospital

_6_ Nursing homes, long-term care facilities, or rehabilitation facilities associated with your hospital

_7_ None of the above

1. Please indicate below if your antimicrobial stewardship program has one or more interventions in place to improve appropriate antibiotic use in any of the following settings: (select all that apply)

_1_ Inpatient units (in hospital)

_2_ Outpatient/ambulatory clinics associated with your hospital

_3_ At the time of discharge from the hospital

_4_ Emergency room(s)

_5_ Urgent care facilities associated with your hospital

_6_ Nursing homes, long-term care facilities, or rehabilitation facilities associated with your hospital

_7_ None of the above

1. Does your facility provide tele-stewardship (i.e., using remote monitoring to help other facilities)?

_1_ No

_2_ Yes

_3_ Don’t know

1. Does your facility receive tele-stewardship (i.e., another group helps you monitor antibiotic use)?

_1_ No

_2_ Yes

_3_ Don’t know

1. Does your hospital have any strategies in place to reduce inappropriate testing *for any of the following*? (select all that apply)

_1_ Urine testing (including urine cultures or urinalyses)

_2_ Blood cultures

_3_ Respiratory cultures

_4_ Respiratory panel by PCR

_5_ Stool gastrointestinal pathogen panel by PCR

1. At your hospital, contact precautions for MRSA are required for which of the following: (select only one)

_1_ All MRSA colonized or infected patients and we do MRSA surveillance culturing

_2_ All MRSA colonized or infected patients, but we do NOT do MRSA surveillance culturing

_3_ MRSA infected patients only

_4_ MRSA colonized or infected patients under special circumstances (e.g., open wounds)

_5_ Not required

1. At your hospital, contact precautions for vancomycin-resistant enterococci (VRE) are required for which of the following: (select only one)

_1_ All VRE colonized or infected patients and we do VRE surveillance culturing

_2_ All VRE colonized or infected patients, but we do NOT do VRE surveillance culturing

_3_ VRE infected patients only

_4_ VRE colonized or infected patients under special circumstances (e.g., open wounds)

_5_ Not required

1. At your hospital, contact precautions for carbapenem-resistant Enterobacteriaceae (CRE) are required for which of the following: (select only one)

_1_ All CRE colonized or infected patients and we do CRE surveillance culturing

_2_ All CRE colonized or infected patients, but we do NOT do CRE surveillance culturing

_3_ CRE infected patients only

_4_ CRE colonized or infected patients under special circumstances (e.g., open wounds)

_5_ Not required

1. The following pertain to adults hospitalized in your acute care facility.

| **Infection prevention practice** | Using a scale from 1 to 5  (1 being never and 5 being always), please indicate **how** **frequently the practice is used** in your hospital |
| --- | --- |
| A. Decolonization of the nose and skin in patients colonized with methicillin-resistant *Staphylococcus aureus* (MRSA) prior to a surgical procedure | 1 2 3 4 5  Never Always |
| B. Chlorhexidine gluconate for daily bathing of ICU patients | 1 2 3 4 5  Never Always |
| C. Chlorhexidine gluconate for daily bathing of non-ICU patients | 1 2 3 4 5  Never Always |

1. The following questions pertain to annual influenza vaccination of healthcare workers at your hospital.
   1. Does your hospital mandate healthcare workers to receive annual influenza vaccination? (select only one)

_1_ No hospital mandates regarding influenza vaccination

_2_ Healthcare workers are encouraged to get vaccination, but it is not mandated

_3_ There is a mandate requiring healthcare workers to get vaccination, with the option of opting out for allowable reasons

_4_ Don’t know

- 1. Which of the following are allowable reasons for healthcare workers to opt out or decline vaccination at your hospital? (select all that apply)

_1_ Medical contraindication

_2_ Religious reasons

_3_ No reason required

_4_ Other, please specify: ___________________________________________________

_5_ Not applicable / annual influenza vaccination not mandated

- 1. Are those who do not receive annual influenza vaccination required to wear a mask when providing patient care during the flu season?

_1_ No

_2_ Yes

_3_ Don’t know

- 1. Are there any penalties for healthcare workers who are non-compliant with your facility’s policy on influenza vaccination?

_1_ No

_2_ Yes, please specify: _______________________________________________

_3_ Don’t know

# *COVID-19:*The following questions pertain to your hospital’s experiences during the COVID-19 pandemic at any point from March 2020 until the present.

1. In your opinion, how effective has your hospital’s pandemic response plan been in addressing COVID-19?

_1_ Minimally effective

_2_ Moderately effective

_3_ Very effective

_4_ Extremely effective

_5_ Not applicable, our hospital did not have a pandemic response plan

1. Which organization has your facility relied on the most for information about COVID-19? (select only one)

_1_ Israel Ministry of Health

_2_ Centers for Disease Control & Prevention (CDC)

_3_ World Health Organization (WHO)

_4_ Centers for Medicare & Medicaid Services (CMS)

_5_ Infectious Diseases Society of America (IDSA)

_6_ Association for Professionals in Infection Prevention & Epidemiology (APIC)

_7_ State and/or local health department

_8_ Local hospital/healthcare organization

_9_ Other, please specify: _____________________________________________________________

1. Has your hospital designated areas to care for COVID-19 patients that are separated from non-COVID patients?

_1_  No

_2_ Yes, but only in some units (e.g., ICU)

_3_ Yes, hospital wide

_4_ Don’t know

1. Has your hospital opened new units to care for COVID-19 patients?

_1_  No

_2_ Yes

_3_ Don’t know

1. Has your hospital experienced staff shortages due to absences and/or illness during the COVID-19 pandemic?

_1_ No

_2_ Yes

_3_ Don’t know

1. Has your hospital experienced an increased loss of staff (e.g., resignations) in the midst of COVID-19?

_1_ No

_2_ Yes

_3_ Don’t know

1. Has your hospital experienced a shortage of any supplies during the COVID-19 pandemic? (select all that apply)

_1_ N95 masks

_2_ Powered air-purifying respirators (PAPRs)

_3_ Alcohol-based hand sanitizer

_4_ Gowns

_5_ Gloves

_6_ Surgical masks

_7_ Full face shields

_8_ Eye shields/goggles

_9_ Disinfectant wipes

_10_ Other, please specify:                                         ________________________

_11_ No supply shortages experienced

1. Has your hospital experienced financial hardship resulting from the COVID-19 pandemic?

_1_ No financial hardship

_2_ Mild financial hardship

_3_ Moderate financial hardship

_4_  Extreme financial hardship

_5_  Don’t know

1. Please indicate how much you agree or disagree with the following statement. “I feel safe carrying out my work role during the COVID-19 pandemic?”

_1_ Strongly disagree

_2_ Disagree

_3_ Neither disagree nor agree

_4_ Agree

_5_ Strongly agree

1. How confident are you that a COVID-19 vaccine is safe and effective?

_1_ Not confident at all

_2_ Only slightly confident

_3_ Somewhat confident

_4_  Moderately confident

_5_  Very confident

1. If your employer does not require it, would you or have you voluntarily vaccinated yourself against COVID-19?

_1_ No

_2_ Yes

_3_ Don’t know/Prefer not to answer

1. In your opinion, how effective has your hospital’s COVID-19 vaccination plan been in vaccinating staff?

_1_ Minimally effective

_2_ Moderately effective

_3_ Very effective

_4_ Extremely effective

_5_ Not applicable, our hospital does not have a COVID-19 vaccination plan for staff

***Final questions:*** The following pertain only to the person completing this survey.

1. What is your position in the hospital?

_1_ Infection Preventionist

_2_ Nursing Management

_3_ Hospital Epidemiologist

_4_ Other, please specify: ____________________________________________

1. How long have you had this position?

______ years _______ months

1. How long have you worked at this hospital?

______ years _______ months

**Congratulations! You are finished. Thank you for completing this survey!**

Please provide any additional comments on the next page:
