# Supplementary material for: Healthcare-associated infection prevention and control practices in Israel: results of a national survey
Source: BMC Infect Dis. 2022 Sep 16;22:739. doi: 10.1186/s12879-022-07721-8 (PMC9482296; doi:10.1186/s12879-022-07721-8)
Supplement: Supplementary file 2 — Additional file 2. Results for individual survey questions. [file 12879_2022_7721_MOESM2_ESM.docx]

Supplementary Appendix 2: Individual Survey Responses

Healthcare-associated infection prevention and control practices in Israel: Results of a national survey

**Supplementary Appendix: Tables**

**Contents**

Table S1: Hospital and IPC Staff Characteristics 3

Table S2: CAUTI Practices 4

Table S3: CLABSI Practices 5

Table S4: VAP Practices 6

Table S5: *Clostridioides difficile* IPC Practices 7

Table S6: General IPC Practices 8

Table S7: COVID-19 IPC Practices 9

**Table Notes**:

Bold font represents questions in which percentages represent agree/strongly agree

Answers of ‘don’t know’ were removed

**Abbreviations**:

IPC: Infection prevention and control

CAUTI: Catheter associated urinary tract infection

CLABSI: Central line associated bloodstream infection

VAP: Ventilator associated pneumonia

*C. difficile*: *Clostridioides* *difficile*

COVID-19: Coronavirus disease 2019

**Table S1: Hospital and IPC Staff Characteristics**

| **Question** | **N Response** | **N Yes** | **%** |
| --- | --- | --- | --- |
| Total number of adult acute care beds | 15 |  |  |
| Total number of adult Intensive Care Unit (ICU) beds | 15 |  |  |
| Private rooms | 14 |  |  |
| Non-private rooms | 14 |  |  |
| Med school affiliation | 15 | 15 | 100% |
| Highest ranking physician provides direct patient care | 15 | 8 | 53.3% |
| Highest ranking nurse provides direct patient care | 15 | 5 | 33.3% |
| Very good to excellent support for IP from hospital leadership | 15 | 5 | 33.3% |
| Has hospital epidemiologist | 15 | 11 | 73.3% |
| **I feel burned out from my work** | **15** | **2** | **13.3%** |
| **I have become more uncaring towards people since I took this job.** | **15** | **1** | **6.7%** |
| **If given the opportunity to revisit my career choice, I would choose to become an infection preventionist again.** | **15** | **11** | **73.3%** |
| **Spiritual well-being is important for one’s emotional well-being** | **15** | **12** | **80.0%** |
| **Religious or spiritual beliefs act as a source of comfort and strength during life’s ups and downs** | **15** | **9** | **60.0%** |
| **An organized religious or spiritual community is important to me** | **15** | **6** | **40%** |
| **Individual self-care practices (e.g., meditation, yoga, listening to music, exercising, communing with nature) is important to me** | **15** | **12** | **80%** |
| **I assert my views on important issues, even though my supervisor may disagree** | **15** | **15** | **100%** |
| **I personally feel comfortable speaking up when I see a physician not clean his or her hands.** | **15** | **15** | **100%** |
| **When a medical error occurs at this hospital, employees are encouraged to discuss mistakes in order to learn how to prevent similar future errors.** | **15** | **13** | **86.7%** |
| **Leadership is driving us to be a safety-centered institution** | **15** | **12** | **80%** |
| **I would feel safe being treated here as a patient** | **15** | **10** | **66.7%** |
| **If you make a mistake at this hospital, it is often held against you** | **15** | **1** | **6.7%** |
| **Employees at this hospital are able to bring up problems and tough issues** | **15** | **9** | **60%** |
| **It is safe to try something new at this hospital** | **15** | **13** | **86.7%** |
| **At this hospital, people are too busy to invest time in improvement** | **15** | **4** | **26.7%** |
| **In this hospital, employees are expected to question leadership** | **15** | **5** | **33.3%** |
| **In this hospital, authority is concentrated at the top** | **15** | **8** | **53.3%** |

**Table S2: CAUTI Practices**

| **Question** | **N Response** | **N Yes** | **%** |
| --- | --- | --- | --- |
| **Bladder ultrasound scanner** | **14** | **4** | **28.6%** |
| **Urinary catheter reminder or stop-order** | **14** | **5** | **35.7%** |
| **Nurse-initiated urinary catheter discontinuation** | **14** | **2** | **14.3%** |
| **Silver alloy Foley catheters** | **14** | **1** | **7.1%** |
| **External catheters in men** | **14** | **2** | **14.3%** |
| **External catheters in women** | **14** | **0** | **0%** |
| **Aseptic technique** | **14** | **12** | **85.7%** |
| **Intermittent catheterization** | **14** | **0** | **0%** |
| **Restricted list of appropriate clinical indications to place indwelling urinary catheters** | **14** | **6** | **42.9%** |
| Does your hospital have a system for monitoring which patients have urinary catheters placed (some units or hospital wide) | 15 | 13 | 86.7% |
| Does your hospital have a system for monitoring which patients have urinary catheters placed (some units) | 15 | 3 | 20% |
| Does your hospital have a system for monitoring which patients have urinary catheters placed (hospital wide) | 15 | 10 | 66.7% |
| Where are the majority of indwelling urinary catheters placed at your hospital | 15 |  |  |
| Emergency Department |  | 10 | 66.7% |
| Floor (including telemetry/step down) |  | 2 | 13.3% |
| Intensive Care Unit |  | 3 | 20% |
| Does your hospital conduct daily rounds to assess the ongoing necessity of indwelling urinary catheters (some units or hospital wide) | 15 | 11 | 73.3% |
| Does your hospital conduct daily rounds to assess the ongoing necessity of indwelling urinary catheters (some units) | 15 | 9 | 60% |
| Does your hospital conduct daily rounds to assess the ongoing necessity of indwelling urinary catheters (hospital wide) | 15 | 2 | 13.3% |
| Does your hospital routinely monitor duration and/or discontinuation of urinary catheters (some units or hospital wide) | 15 | 13 | 86.7% |
| Does your hospital routinely monitor duration and/or discontinuation of urinary catheters (some units) | 15 | 9 | 60% |
| Does your hospital routinely monitor duration and/or discontinuation of urinary catheters (hospital wide) | 15 | 4 | 26.7% |
| Does your hospital have an established surveillance system for monitoring urinary tract infection rates (some units or hospital wide) | 15 | 14 | 93.3% |
| Does your hospital have an established surveillance system for monitoring urinary tract infection rates (some units) | 15 | 12 | 80% |
| Does your hospital have an established surveillance system for monitoring urinary tract infection rates (hospital wide) | 15 | 2 | 13.3% |
| Does your hospital report urinary tract infection rates to direct care providers (some units or hospital wide) | 14 | 11 | 78.5% |
| Does your hospital report urinary tract infection rates to direct care providers (some units) | 14 | 9 | 64.2% |
| Does your hospital report urinary tract infection rates to direct care providers (hospital wide) | 14 | 2 | 14.2% |
| Does your hospital report urinary catheter utilization ratios to direct care providers (some units or hospital wide) | 15 | 13 | 86.7% |
| Does your hospital report urinary catheter utilization ratios to direct care providers (some units) | 15 | 11 | 73.3% |
| Does your hospital report urinary catheter utilization ratios to direct care providers (hospital wide) | 15 | 2 | 13.3% |
| What is your perception of how important it is to hospital leadership at your hospital to prevent urinary tract infections | 15 | 5 | 33.3% |
| Does your hospital perform routine urine tests (for example, urinalysis and/or urine culture) to screen for urinary tract infection at the time of hospital admission | 15 | 1 | 6.7% |
| Does your hospital use any of the following strategies to reduce inappropriate urine testing (select all that apply): | 10 |  |  |
| Require indication when placing order for urine test |  | 4 | 40% |
| Encourage appropriate urine testing using decision-support or electronic alerts (e.g., best practice alerts) |  | 1 | 10% |
| Rejecting contaminated urine specimens |  | 10 | 100% |
| Selective suppression of antimicrobial susceptibility results |  | 3 | 30% |
| Reflex urine testing (i.e., only perform urine culture when urinalysis is positive) |  | 2 | 20% |
| Nurse-directed education/initiative to reduce urine testing |  | 8 | 80% |
| Remove/limit urine culture testing |  | 1 | 10% |

**Table S3: CLABSI Practices**

| **Question** | **N Response** | **N Yes** | **%** |
| --- | --- | --- | --- |
| **Maximum sterile barrier precautions** | **14** | **14** | **100%** |
| **Alcohol-containing chlorhexidine gluconate for skin antisepsis at the insertion site** | **14** | **14** | **100%** |
| **Advanced securement device** | **13** | **11** | **84.6%** |
| **Impregnated or antiseptic coated catheters** | **14** | **1** | **7.1%** |
| **Use of cyanoacrylate glue at the exit site** | **14** | **0** | **0%** |
| **Antimicrobial dressing with chlorhexidine (Biopatch™)** | **14** | **6** | **42.9%** |
| **Restricted list of appropriate clinical indications to place central venous catheters** | **14** | **5** | **35.7%** |
| Does your hospital have an established surveillance system for monitoring central venous catheter-related infection rates | 14 | 14 | 100% |
| Does your hospital have an established surveillance system for monitoring central venous catheter-related infection rates (in some units) | 14 | 11 | 78.6% |
| Does your hospital have an established surveillance system for monitoring central venous catheter-related infection rates (hospital wide) | 14 | 3 | 21.4% |
| Does your hospital report central venous catheter-related infection rates to direct care providers | 15 | 13 | 86.7% |
| Does your hospital report central venous catheter-related infection rates to direct care providers (in some units) | 15 | 11 | 73.3% |
| Does your hospital report central venous catheter-related infection rates to direct care providers (hospital wide) | 15 | 2 | 13.3% |
| Who is responsible for inserting the majority of non-peripherally inserted central venous catheters at your hospital (select only one) | 15 |  |  |
| Intensive Care Unit Physicians |  | 12 | 80% |
| Interventional Radiologists |  | 3 | 20% |
| Does your hospital have a list of indications to determine the appropriateness of central venous catheters prior to placement | 15 | 7 | 46.7% |
| Who is responsible for inserting the majority of peripherally inserted central catheters (PICCs) at your hospital | 15 |  |  |
| Emergency Room Physicians |  | 1 | 6.67% |
| Interventional Radiologists |  | 14 | 93.3% |
| Does your hospital have a list of indications to determine the appropriateness of PICCs prior to placement | 15 | 6 | 40% |
| Does your hospital use appropriateness guidelines (e.g., Michigan MAGIC) for PICC use | 14 | 3 | 21.4% |
| Does your hospital conduct daily rounds to assess the ongoing necessity of PICCs | 15 | 7 | 46.7% |
| Does your hospital conduct daily rounds to assess the ongoing necessity of PICCs – some units | 15 | 6 | 40% |
| Does your hospital conduct daily rounds to assess the ongoing necessity of PICCs – hospital wide | 15 | 1 | 6.7% |
| What is your perception of how important it is to hospital leadership at your hospital to prevent central venous catheter-related infections | 15 | 12 | 80% |

**Table S4: VAP Practices**

| **Question** | **N Response** | **N Yes** | **%** |
| --- | --- | --- | --- |
| **Semi-recumbent positioning** | **14** | **13** | **92.9%** |
| **Antimicrobial mouth rinse** | **14** | **13** | **92.9%** |
| **Subglottic secretion drainage** | **14** | **3** | **21.4%** |
| **Topical and/or systemic antibiotics for selective digestive tract decontamination** | **14** | **0** | **0%** |
| **Silver-coated endotracheal tube** | **14** | **0** | **0%** |
| **Sedation vacation** | **14** | **4** | **28.6%** |
| Does your hospital encourage early mobilization of ventilated patients as a strategy to prevent ventilator-associated events | 15 | 8 | 53.3% |
| Does your hospital encourage early mobilization of ventilated patients as a strategy to prevent ventilator-associated events - in some units | 15 | 6 | 40.0% |
| Does your hospital encourage early mobilization of ventilated patients as a strategy to prevent ventilator-associated events- hospital wide | 15 | 2 | 13.3% |
| Does your hospital have an established surveillance system for monitoring ventilator-associated event rates | 14 | 6 | 42.9% |
| Does your hospital have an established surveillance system for monitoring ventilator-associated event rates – some units | 14 | 5 | 35.7% |
| Does your hospital have an established surveillance system for monitoring ventilator-associated event rates- hospital wide | 14 | 1 | 7.1% |
| Does your hospital report ventilator-associated event rates to direct care providers | 14 | 5 | 35.7% |
| Does your hospital report ventilator-associated event rates to direct care providers – some units | 14 | 5 | 35.7% |
| Does your hospital report ventilator-associated event rates to direct care providers – hospital wide | 14 | 0 | 0% |
| What is your perception of how important it is to hospital leadership at your hospital to prevent ventilator-associated events | 15 | 3 | 20% |

**Table S5: *Clostridioides* *difficile* IPC Practices**

| **Question** | **N Response** | **N Yes** | **%** |
| --- | --- | --- | --- |
| Does your hospital use supplemental no-touch disinfection devices for rooms used to care for patients with *C. difficile* infection | 15 | 8 | 53% |
| Does your hospital use supplemental no-touch disinfection devices for rooms used to care for patients with *C. difficile* infection -some units | 15 | 3 | 20% |
| Does your hospital use supplemental no-touch disinfection devices for rooms used to care for patients with *C. difficile* infection – hospital wide | 15 | 5 | 33.3% |
| Does your hospital use real-time methods to assess thoroughness of cleaning and disinfection of environmental surfaces in patient rooms | 15 | 15 | 100% |
| Does your hospital use real-time methods to assess thoroughness of cleaning and disinfection of environmental surfaces in patient rooms – some units | 15 | 2 | 13.3% |
| Does your hospital use real-time methods to assess thoroughness of cleaning and disinfection of environmental surfaces in patient rooms – hospital wide | 15 | 13 | 86.7% |
| Does your hospital have a written policy to routinely test for *C. difficile* when patients have diarrhea while on antibiotics or within several months of taking them | 15 | 11 | 73.3% |
| Does your hospital have a written policy to routinely test for *C. difficile* when patients have diarrhea while on antibiotics or within several months of taking them – some units | 15 | 0 | 0% |
| Does your hospital have a written policy to routinely test for *C. difficile* when patients have diarrhea while on antibiotics or within several months of taking them – hospital wide | 15 | 11 | 73.3% |
| Does your hospital have an established surveillance system for monitoring *C. difficile* infection rates | 15 | 15 | 100% |
| Does your hospital have an established surveillance system for monitoring *C. difficile* infection rates – some units | 15 | 0 | 0% |
| Does your hospital have an established surveillance system for monitoring *C. difficile* infection rates – hospital wide | 15 | 15 | 100% |
| Are clinicians at your hospital educated as to when to order *C. difficile* testing | 15 | 14 | 93.3% |
| Which test does your laboratory primarily use to determine the presence of C. difficile (select one) | 15 |  |  |
| Polymerase chain reaction (PCR) |  | 3 | 20% |
| Some combination of the above |  | 12 | 80% |
| Does your hospital use any of the following strategies to reduce inappropriate testing for *C. difficile* (select all apply) |  |  |  |
| Discourage testing for C. difficile on patients on laxatives using decision-support or electronic alerts (e.g., best practice alerts) |  | 3 | 21.4% |
| Other, please specify: |  | 2 | 14.3% |
| Reject formed stool submitted for C. difficile testing |  | 9 | 64.3% |
| Does your hospital offer Fecal Microbiota Transplant (FMT) for patients with recurrent *C. difficile* infection | 15 | 8 | 53.3% |
| Does your hospital report *C. difficile* infection rates to direct care providers | 15 | 14 | 93.3% |
| Does your hospital report *C. difficile* infection rates to direct care providers – some units | 15 | 1 | 6.7% |
| Does your hospital report *C. difficile* infection rates to direct care providers – hospital wide | 15 | 13 | 86.7% |
| What is your perception of how important it is to hospital leadership at your hospital to prevent *C. difficile* infection | 15 | 10 | 66.7% |

**Table S6: General IPC Practices**

| **Question** | **N Response** | **N Yes** | **%** |
| --- | --- | --- | --- |
| Do you feel you are under any pressure to NOT report healthcare-associated infections at your hospital | 15 | 1 | 6.7% |
| Among all patient safety issues, how important is hand hygiene at your hospital | 15 | 15 | 100% |
| Hand hygiene compliance | 15 | 14 | 83.1% |
| Which surveillance method(s) does your hospital use for monitoring hand hygiene compliance (select all that apply) |  |  |  |
| Direct observation method by validated observer | 15 | 15 | 100% |
| Does your hospital have an antimicrobial stewardship program | 15 | 15 | 100% |
| Does your facility provide tele-stewardship | 15 | 3 | 20% |
| Does your facility receive tele-stewardship | 15 | 1 | 6.7% |
| Decolonization of the nose and skin in patients colonized with methicillin-resistant *Staphylococcus aureus* (MRSA) prior to a surgical procedure | 14 | 4 | 28.6% |
| Chlorhexidine gluconate for daily bathing of ICU patients | 14 | 13 | 92.9% |
| Chlorhexidine gluconate for daily bathing of non-ICU patients | 14 | 3 | 21.4% |
| Does your hospital mandate healthcare workers to receive annual influenza vaccination | 15 |  |  |
| Healthcare workers are encouraged to get vaccination, but it is not mandated |  | 14 | 93.3% |
| There is a mandate requiring healthcare workers to get vaccination, with the option of opting out for allowable reasons |  | 1 | 6.7% |
| Which of the following are allowable reasons for healthcare workers to opt out or decline vaccination at your hospital |  |  |  |
| Medical contraindication |  | 1 | 6.6% |
| No reason required |  | 4 | 26.6% |
| Not applicable / annual influenza vaccination not mandated |  | 9 | 60% |
| Other, please specify: ideological reasons |  | 1 | 6.6% |
| Are those who do not receive annual influenza vaccination required to wear a mask when providing patient care during the flu season | 15 | 1 | 6.7% |
| Are there any penalties for healthcare workers who are non-compliant with your facility’s policy on influenza vaccination | 15 | 0 | 0% |

**Table S7: COVID-19 IPC Practices**

| **Question** | **N Response** | **N Yes** | **%** |
| --- | --- | --- | --- |
| In your opinion, how effective has your hospital’s pandemic response plan been in addressing COVID-19 | 15 | 12 | 80% |
| Which organization has your facility relied on the most for information about COVID-19 (select one) | 14 |  |  |
| Centers for Disease Control & Prevention (CDC) |  | 2 | 14.3% |
| Israel Ministry of Health |  | 11 | 78.6% |
| Local hospital/healthcare organization |  | 1 | 7.1% |
| Has your hospital designated areas to care for COVID-19 patients that are separated from non-COVID patients | 15 | 15 | 100% |
| Has your hospital designated areas to care for COVID-19 patients that are separated from non-COVID patients – in some units | 15 | 1 | 6.7% |
| Has your hospital designated areas to care for COVID-19 patients that are separated from non-COVID patients – hospital wide | 15 | 14 | 93.3% |
| Has your hospital opened new units to care for COVID-19 patients | 15 | 15 | 100% |
| Has your hospital experienced staff shortages due to absences and/or illness during the COVID-19 pandemic | 15 | 11 | 73.3% |
| Has your hospital experienced an increased loss of staff (e.g., resignations) in the midst of COVID-19 | 15 | 6 | 40% |
| Has your hospital experienced a shortage of any supplies during the COVID-19 pandemic |  |  |  |
| N95 masks |  | 8 | 53.3% |
| Powered air-purifying respirators (PAPRs) |  | 1 | 6.6% |
| Alcohol-based hand sanitizer |  | 4 | 26.6% |
| Gowns |  | 5 | 33.3% |
| Gloves |  | 3 | 20% |
| Surgical masks |  | 3 | 20% |
| Full face shields |  | 3 | 20% |
| Disinfectant wipes |  | 2 | 13.3% |
| Only in the beginning of the pandemic |  | 1 | 6.6% |
| No supply shortages experienced |  | 2 | 20% |
| Has your hospital experienced financial hardship resulting from the COVID-19 pandemic | 10 | 10* | 100% |
| Please indicate how much you agree or disagree with the following statement. “I feel safe carrying out my work role during the COVID-19 pandemic | 15 | 14 | 93.3% |
| How confident are you that a COVID-19 vaccine is safe and effective | 15 | 15 | 100% |
| If your employer does not require it, would you or have you voluntarily vaccinated yourself against COVID-19 | 15 | 15 | 100% |
| In your opinion, how effective has your hospital’s COVID-19 vaccination plan been in vaccinating staff | 15 | 15 | 100% |

* Response breakdown: 8 (moderate/extreme); 2 (mild)
